# *Erratum*: Treatment of Dystrophic *mdx* Mice with an ADAMTS-5 Specific Monoclonal Antibody Increases the Ex Vivo Strength of Isolated Fast Twitch Hindlimb Muscles. *Biomolecules* 2020, *10*, 416

**DOI:** 10.3390/biom10071053

**Published:** 2020-07-15

**Authors:** Alex B. Addinsall, Leonard G. Forgan, Natasha L. McRae, Rhys W. Kelly, Penny L. McDonald, Bryony McNeill, Daniel R. McCulloch, Nicole Stupka

**Affiliations:** 1 Centre for Molecular and Medical Research, School of Medicine, Deakin University, Waurn Ponds, VIC 3216, Australia; alex.addinsall@ki.se (A.B.A.); leonard.forgan@deakin.edu.au (L.G.F.); natasha.mcrae1@deakin.edu.au (N.L.M.); rwkelly@deakin.edu.au (R.W.K.); penny.mcdonald@mcri.edu.au (P.L.M.); bryony.mcneill@deakin.edu.au (B.M.); daniel.mcculloch@uq.net.au (D.R.M.); 2 Department of Physiology and Pharmacology, Karolinska Insitutet, 171 77 Stockholm, Sweden; 3 Department of Medicine—Western Health, The University of Melbourne, St. Albans, VIC 3021, Australia; 4 Australian Institute for Musculoskeletal Science (AIMSS), 176 Furlong Road, St. Albans, VIC 3021, Australia

The authors wish to make a change to this published paper [1]. In the original manuscript, there was a mistake in the labeling of the y-axis for Figure 5B,D. To assess muscle fatiguability, extensor digitorum longus (EDL) and soleus muscles were subjected to 4 min of intermittent (1 contraction every 5 s), submaximal stimulation at 60 Hz. The force output of every fifth contraction is statistically presented as a data point. The revision to Figure 5B,D now reflects the correct stimulation number.

The authors apologize for any inconvenience caused, and we wish to stress that this change does not affect the presented scientific results and statistical analyses. The manuscript with be updated and the original will remain online on the article webpage at https://www.mdpi.com/2218-273X/10/3/416.

## Figures and Tables

**Figure 5:**
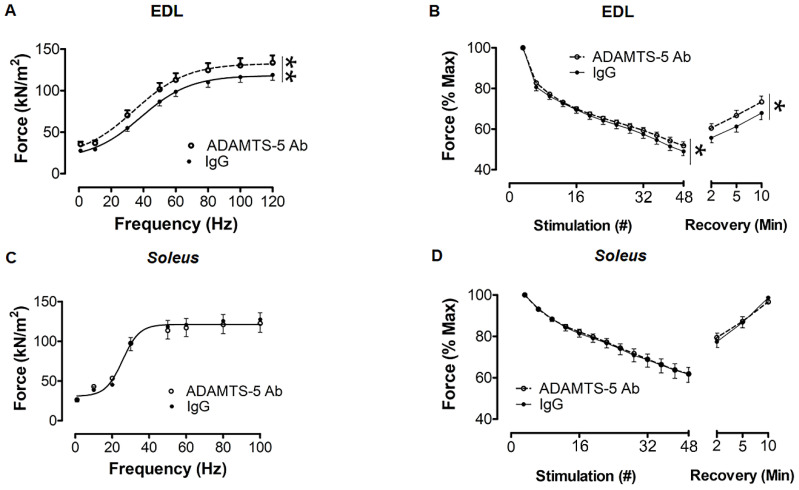
In *mdx* mice, ADAMTS-5 blockade improved the contractile function of fast EDL muscles. (**A**) Treatment with the ADAMTS-5 mAb increased the specific isometric force (sP_o_) output of EDL muscles. (**B**) During 4 min of intermittent 60 Hz stimulation, the relative fatigability of EDL muscles from *mdx* mice treated with the ADAMTS-5 mAb was reduced, and the relative force recovery was also improved. In *soleus* muscles, ADAMTS-5 blockade had no significant effect on (**C**) isometric strength or (**D**) fatigability and force recovery. * *p* < 0.05 and ** *p* < 0.001, main effect treatment, 2-way GLM-ANOVA. *N* = 17–19 mice.

## References

[1] Addinsall A.B., Forgan L.G., McRae N.L., Kelly R.W., McDonald P.L., McNeil B., McCulloch D.R., Stupka N. (2020). Treatment of Dystrophic *mdx* Mice with an ADAMTS-5 Specific Monoclonal Antibody Increases the Ex Vivo Strength of Isolated Fast Twitch Hindlimb Muscles. Biomolecules.

